# Mortality-Related Factors and 1-Year Survival in Patients After Intracranial Stenting for Intracranial Arterial Critical Stenosis and Occlusion

**DOI:** 10.3390/medicina61030404

**Published:** 2025-02-26

**Authors:** Yusuf Inanc, Esra Polat, Mesut Karatas, Cengiz Sabanoglu, Kader Eliz Sahin, Ibrahim Halil Inanc

**Affiliations:** 1 Department of Neurology, University of Gaziantep, Gaziantep 27310, Turkey; drinancccc@gmail.com; 2 Department of Cardiology, Gaziantep City Hospital, Gaziantep 27470, Turkey; 3 Department of Cardiology, Kartal Kosuyolu High Speciality Traning and Research Hospital, Istanbul 34718, Turkey; mesut.cardio@gmail.com; 4 Department of Cardiology, Umraniye Training and Research Hospital, Istanbul 34760, Turkey; drchingiz23@gmail.com; 5 Department of Cardiology, Kocaeli City Hospital, Kocaeli 41060, Turkey; kesahin@yahoo.com; 6 Department of Cardiology, Kırıkkale Yuksek Ihtisas Hospital, Kırıkkale 71100, Turkey; dr.ibrahimhalilinanc@outlook.com

**Keywords:** intracranial stent, stenosis, stroke, mortality, atherosclerosis

## Abstract

*Background*: Studies analyzing factors associated with mortality after intracranial stenting are limited. We aimed to investigate potential factors associated with 1-year mortality after urgent or elective intracranial stenting in those patients with intracranial atherosclerotic stenosis. *Methods*: Patients, who underwent urgent intracranial stenting of the target lesion either due to acute stroke unresponsive to mechanical thrombectomy, or who underwent elective stenting for symptomatic intracranial atherosclerotic stenosis were included in the study. The Modified Rankin Scale (mRS) score was evaluated on admission and grouped accordingly: ≤2 vs. >2. Restenosis and mortality rates in the 1-year follow-up were also analyzed. *Results*: A total of 60 patients were included in the study; the mean age was 60.2 (±10.8). The ratio of urgent/elective intracranial stenting was 7/53. Complete revascularization was achieved in all patients, but no periprocedural complications occurred. The rate of in-hospital mortality was 1/60, 1-year mortality due to any cause 4/60, and restenosis in a 1-year follow-up was 4/60. The age over 65 years, previous history of stroke, atrial fibrillation (AF), and rheumatic mitral valve disease were associated with mortality (*p* < 0.001, *p* = 0.002, *p* = 0.017, and *p* = 0.003, respectively). The median mRS score on admission was lower in the surviving patients at 1 year (*p* = 0.001). *Conclusions*: Intracranial stenting may provide long-term survival with low adverse event rates in elective and selected emergency cases. Advanced age, poor functional status, previous stroke, AF, and rheumatic mitral valve disease are associated with 1-year mortality.

## 1. Introduction

Reperfusion therapy maintaining cerebral blood flow is the main approach in ischemic stroke to rescuing ischemic tissue before infarction. The benefit of reperfusion therapy diminishes with time; hence, prompt management is necessary, with mechanical thrombectomy and intravenous thrombolytic therapy comprising the acute reperfusion strategies. Rescue intracranial stenting may be performed in emergency settings in the event of an unsuccessful thrombectomy [1]. Intracranial stenting may also be an option in those symptomatic patients with atherosclerotic disease of the intracranial arteries [2].

While earlier randomized controlled trials, such as the SAMMPRIS [3] and VISSIT [4] trials, demonstrated the superiority of aggressive medical management over endovascular stent implantation due to higher periprocedural risks associated with stenting, advancements in endovascular technology and techniques have the potential to improve outcomes [5].

To date, previous studies have used several severity and disability scales such as the NIHSS (the National Institutes of Health Stroke Scale) [6]. The Modified Rankin Scale (mRS) is the most broadly used scale for stroke, which measures functional independence across seven grades [7]. mRS may also be evaluated at 90 days after endovascular or intravenous interventions [8].

A recent study showed that a higher mRS score at discharge was an independent predictor of poor functional outcomes at 90 days. Specifically, patients with a discharge mRS score between 3 and 5 had more than three times the odds of experiencing poor outcomes at 90 days compared to those with lower discharge mRS scores [9]. Similarly,, the mRS score was found to be associated with acute thrombosis in patients with acute stroke after emergent angioplasty or stenting for underlying intracranial stenosis [1].

In this study, we aimed to investigate the potential factors associated with 1-year mortality after intracranial stenting in those patients with acute stroke in whom mechanical thrombectomy was unsuccessful, or in those with symptomatic intracranial atherosclerotic arterial stenosis.

## 2. Methods

This retrospective cohort study was performed in Kırıkkale Yuksek Ihtisas and Gaziantep University Hospitals, and approved by the local Ethics Committee of Kırıkkale University Hospital with approval number 2022.05.16. The study was according to the ethical standards laid down in the 1964 Declaration of Helsinki and its later amendments. Informed written consent was provided from all of the participants.

The adult patients, who underwent urgent intracranial stenting for acute stroke unresponsive to mechanical thrombectomy, or who underwent elective stenting for symptomatic intracranial atherosclerotic arterial stenosis in Neurology Clinics in Gaziantep University Hospital between January 2018 and January 2021 were analyzed, retrospectively. A diagnosis and endovascular treatment of acute stroke and symptomatic intracranial atherosclerotic arterial stenosis was made based on the patient’s clinical and radiological background and based on the previous guidelines [10]. The patients with etiological factors other than atherosclerosis, such as arterial dissection, fibromuscular dysplasia, and focal neurological deficit not correlated with intracranial stenosis were excluded. Those with hemorrhagic stroke, or for whom data were missing were excluded.

Urgent intracranial stenting was considered in patients with acute ischemic stroke due to large vessel occlusion and (i) Persistent intracranial arterial occlusion despite multiple thrombectomy attempts; (ii) Poor or inadequate recanalization (e.g., TICI 0–2a) after thrombectomy; (iii) Recurrent stroke or transient ischemic attacks (TIA) despite optimal medical therapy; (iv) High-grade stenosis (>70%) with hemodynamic compromise, leading to ongoing ischemia; (v) Evidence of thrombus formation or vessel collapse post-thrombectomy; (vi) High-risk for re-occlusion due to underlying atherosclerotic plaque instability; (vii) When thrombectomy is not feasible due to vessel tortuosity or distal location of the clot.

Urgent intracranial stenting was not preferred in patients with (i) Large infarct burden on Alberta Stroke Program Early CT Score (ASPECTS ≤ 5); (ii) Uncontrolled hypertension or bleeding risk; (iii) High risk of hemorrhagic transformation; (iv) Terminal illness, advanced dementia, or severe disability; (v) Contraindications to antiplatelet therapy; (vi) Unfavorable vascular anatomy.

The Modified Rankin Scale (mRS) score was evaluated upon admission and analyzed. The patients were grouped based on mRS on admission: ≤2 vs. >2. On admission, the NIHSS score was also evaluated for patients with acute stroke.

The patients were pretreated with clopidogrel loading, and then, with a maintenance dose. Tirofiban was used in ‘bailout’ situations. The patients were grouped according to the type of aortic arch as previously defined: Type I, Type II, and Type III [11].

**Figure 1:**
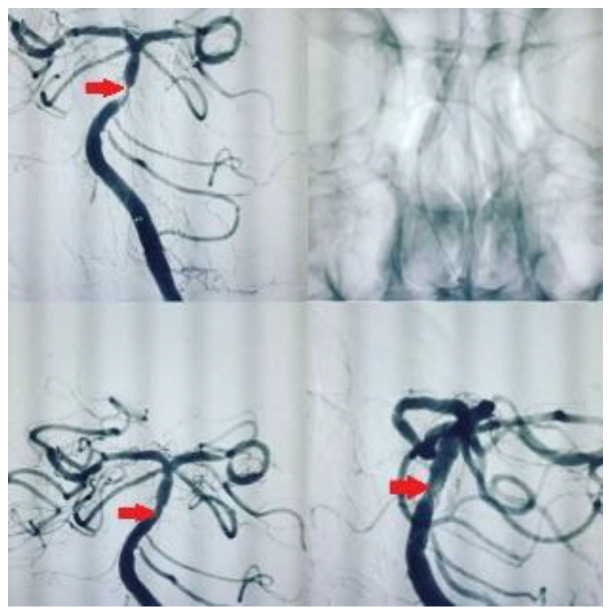
Symptomatic atherosclerotic stenosis in the basilar artery in a 60-year-old male patient, with a history of smoking, type 2 diabetes mellitus, and hypertension, was treated with elective intracranial stenting.

**Figure 2:**
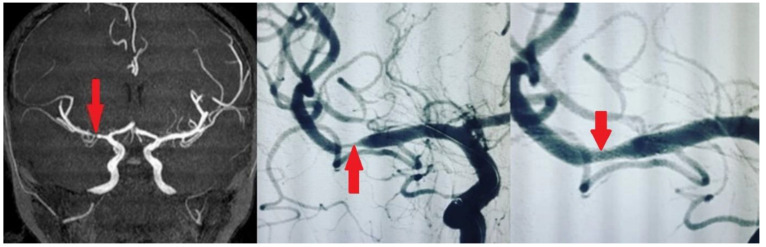
Symptomatic atherosclerotic stenosis in the middle cerebral artery M1 branch in a 57-year-old male patient, with a history of type 2 diabetes mellitus and hyperlipidemia, was treated with elective intracranial stenting.

We used self-expanding stents (Enterprise^®^ stent or ACCLINO^®^ flex plus Stent). The responsible physician selected those stents, which were the best fit for each patient. Angiography was performed with an interventional system (Siemens Axiom Artis zee 2011; Siemens Healthcare, Erlangen, Germany), and predilatation with a balloon was performed in most patients. Examples of stenting in our patients are shown in Figure 1 and Figure 2. We used the Thrombolysis in Cerebral Infarction (TICI) scale to assess the degree of reperfusion following intracranial stenting. It classifies blood flow restoration into five categories based on angiographic findings. TICI 0 represents no perfusion, indicating complete occlusion with no distal flow. TICI 1 signifies minimal perfusion, where some contrast passes beyond the occlusion but without significant tissue perfusion. TICI 2a refers to less than 50% reperfusion of the affected vascular territory, whereas TICI 2b indicates more than 50% but incomplete reperfusion. The highest grade, TICI 3, reflects full perfusion, where the treated vessel achieves complete restoration of blood flow. We accepted TICI 2b–3 as successful revascularization, as it correlates with better functional outcomes and reduced long-term disability.

After the intervention, we observed the clinical signs and symptoms of the patients, and also whether symptomatic intracranial hemorrhage, stent occlusion, or restenosis had occurred or not during hospitalization.

The duration of admission to the intensive care unit (ICU), or hospitalization (days) was also analyzed. In-hospital mortality was defined as death during hospitalization after the intervention. Mortality was defined as death due to any cause in the 1-year follow-up after hospital discharge.

In the 1-year follow-up of the patients, routine transcranial Doppler sonography was performed in all patients, and the decision concerning control magnetic resonance angiography (MRA) or digital subtraction angiography (DSA) was based on the clinical signs or symptoms of ischemia. If any symptoms or signs of ischemia occurred, or anything that was clinically suspect such as uncontrolled hyperglycemia, the stent was controlled by MRA or DSA. Data with respect to restenosis in the 1-year follow-up were obtained for all patients.

## 3. Statistical Analysis

The data were statistically analyzed with SPSS 26.0 (IBM Corporation, Armonk, NY, USA). The normal distribution of the data was tested using the Shapiro–Wilk Francia test. To compare two independent groups of quantitative data according to each other, we used the Mann–Whitney U test with Monte Carlo results. Fisher Exact and the Fisher–Freeman–Halton tests with the Monte Carlo simulation technique were used to compare categorical variables with each other. Comparisons of column proportions were reported as Benjamini–Hochberg-corrected *p*-values. In the tables, quantitative variables were given as mean (standard deviation) and median (minimum–maximum) values, and categorical variables as number (*n*) and percentage (%). We performed receiver operating characteristic (ROC) curve analysis to assess the predictive ability of mRS scores for 1-year mortality. We conducted Kaplan–Meier survival analysis to evaluate 1-year mortality based on mRS scores. Variables were taken into consideration at a 95% confidence level, and a value of *p* < 0.05 was accepted as statistically significant.

## 4. Results

A total of 60 patients were involved in the study. The mean age was 60.2 (±10.8) years, with a median of 63 years (range: 35–81), and the mean BMI was 29.1 (±3.8) kg/m^2^. The most common target lesion locations were the left vertebral artery (31.7%) and the basilar artery (28.3%), followed by the right ICA (13.3%), right MCA (10%), left MCA (8.3%), and right PCA (1.7%). The mean stenosis diameter was 3.9 mm (±0.5), and the mean stenosis length was 22.9 mm (±5.5). The amount of contrast media used had a mean of 246.2 mL (±34.3). The median mRS score on admission was 2 (range: 1–5), while the NIHSS score, available for the seven patients with acute stroke, had a median of 14 (range: 10–20). The median ICU stay was 1 day (range: 1–30), and the median hospitalization duration was 4 days (range: 2–30) (Table 1).

**Figure 3:**
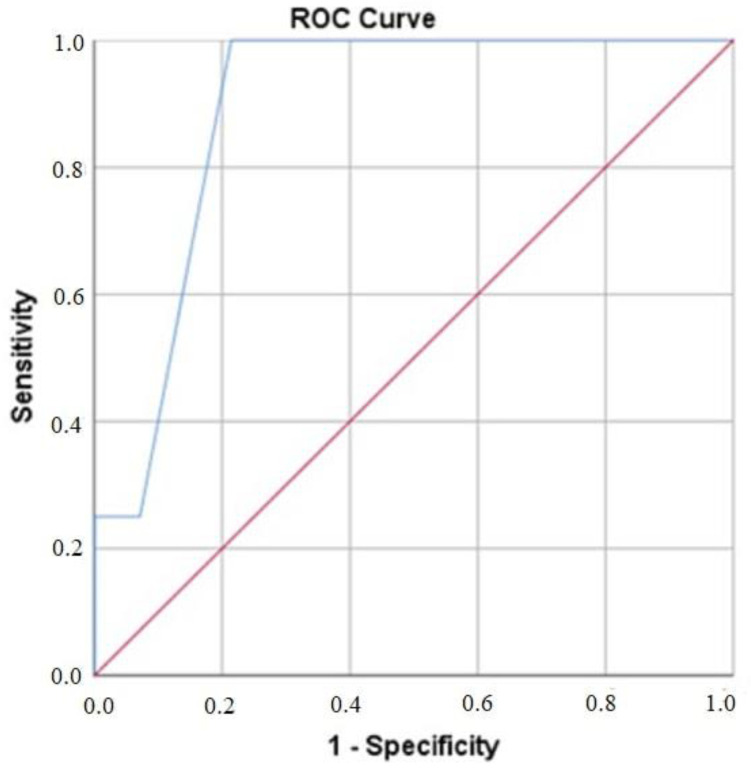
ROC curve analysis of modified Rankin scale score in predicting 1-year mortality.

Table 2 presents the categorical baseline and follow-up characteristics of the patients. Among the 60 patients, 42 (70%) were aged ≤65 years, while 18 (30%) were older than 65. Males comprised 58.3% of the cohort, and 51.7% of patients were smokers. Hypertension (56.7%) and hyperlipidemia (55%) were the most prevalent comorbidities, followed by type 2 diabetes (45%), coronary artery disease (16.7%), heart failure (13.3%), atrial fibrillation (18.3%), and rheumatic mitral valve disease (3.3%). A prior history of stroke was noted in 10% of patients. Most patients (88.3%) underwent elective stenting, while 11.7% required urgent intervention. The majority (73.3%) had an mRS score ≤ 2 at admission, whereas 26.7% had a score > 2. The femoral artery was the primary access site in 88.3% of cases, and predilatation was performed in 95%. Restenosis at one year occurred in 6.7% of patients, while the overall one-year mortality rate was 6.7%, with deaths attributed to major stroke (3.3%) and myocardial infarction (3.3%). In-hospital mortality was recorded in 1.7% of cases (Figure 3).

Table 3 compares clinical parameters between survivors and non-survivors at the one-year follow-up. Patients who died were significantly older, with a median age of 78.5 years compared to 62 years in survivors (*p* < 0.001), and all non-survivors were older than 65 (*p* < 0.001). A higher proportion of non-survivors had a history of stroke (75% vs. 5.4%, *p* = 0.002), atrial fibrillation (75% vs. 14.3%, *p* = 0.017), and rheumatic mitral valve disease (50% vs. 0%, *p* = 0.003). Functional status on admission was worse in non-survivors, with all having an mRS score > 2 compared to 21.4% of survivors (*p* = 0.004), and their median mRS score was significantly higher (*p* = 0.001). Other factors, including sex, smoking, hypertension, diabetes, heart failure, coronary artery disease, hyperlipidemia, site of stenosis, urgency of intervention, restenosis, and procedural details, did not differ significantly between groups. The median ICU stay and hospitalization duration were also similar between survivors and non-survivors.

**Figure 4:**
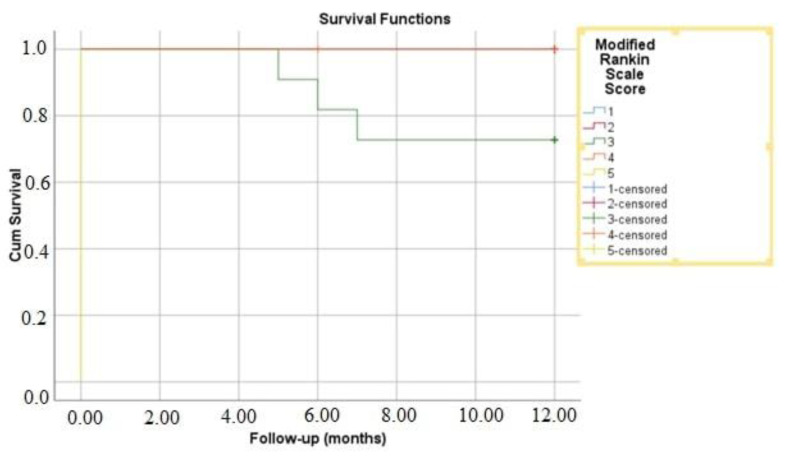
Kaplan–Meier survival analysis of 1-year mortality based on modified Rankin scale. score.

The ROC curve analysis was performed to determine the predictive value of the mRS score at admission for 1-year mortality. The optimal cutoff value was identified as 2.5, yielding a sensitivity of 100% and a specificity of 78.6% with the AUC value of 0.89 (95% CI: 0.796–0.989, *p* = 0.009) (Figure 4).

## 5. Discussion

Our study has the following findings: (i) Death and restenosis in the 1-year follow-up occurred in a minority of patients (6.6%). (ii) Older age, a higher mRS score on admission, previous history of stroke, AF, and rheumatic mitral valve disease were associated with 1-year mortality. (iii) Critical stenosis in VA, ICA, or urgency of stenting was not associated with 1-year mortality.

Two major randomized controlled trials, Stenting and Aggressive Medical Management for Preventing Recurrent Stroke (SAMMPRIS) Trial and the Vitesse Intracranial Stent Study for Ischemic Stroke Therapy (VISSIT), revealed negative results such as a higher rate of stroke and mortality with intracranial stenting compared to medical therapy [3,4]. These findings proposed that medical treatment be the standard of care for patients with symptomatic intracranial atherosclerotic stenosis. However, studies conducted on intracranial stenting in those patients with intracranial atherosclerotic stenosis have gained popularity in the last decade, and revealed new perspectives. Ma et al. [12] included 287 symptomatic patients with intracranial atherosclerotic stenosis, who had received either a balloon-mounted stent or a balloon plus self-expanding stent, and showed that the probability of mortality at 1 year was 1.1%, and restenosis 25.7%. We showed a higher 1-year mortality rate, but a lower frequency of restenosis, however, we included those patients, who had undergone both urgent and elective stenting. Another multicenter study investigated 1-month outcomes after intracranial stenting was performed 3 weeks after an index ischemic event [13]. Target lesions were found mostly in MCA, the intracranial ICA, the intradural VA, and the basilar artery, and the 1-month stroke rate was 2%, but no deaths were observed. They showed that the median mRS score on admission was 0. We showed an in-hospital mortality rate of 1.7%, but no restenosis was detected during hospitalization. In our study, more than a quarter of the participants had an mRS score on the admission of >2, with a higher mRS score associated with mortality. The patients included in our study might have had less functional independence in the preadmission period.

Two recent studies by Ma et al. [14] and Ueda et al. [15] reported procedural success rates of 100% suggesting that the procedural outcomes in their studies align with the high success rate observed in our study, highlighting the effectiveness of current treatment strategies and novel techniques, which make revascularization procedures more efficient and easier to achieve.

Studies analyzing the factors associated with 1-year mortality following intracranial stenting are still limited. When comparing our study with other studies [1,15,16,17], several important similarities and differences emerge, particularly in terms of procedural success, long-term outcomes, restenosis rates, mortality rates, functional status as measured by mRS scores, and lesion characteristics. Stracke et al. [1] reported high procedural success in their cohort of acute stroke patients undergoing emergency intracranial stenting, but their study primarily focused on early outcomes, with no direct comparison to medical management concerning long-term mortality or functional status. While early adverse events were observed at a similar frequency in both the stenting and medical groups, they did not provide long-term follow-up data on outcomes such as restenosis rates or mRS scores. Moreover, lesion characteristics were not specifically discussed. In contrast, our study observed a restenosis rate of 6.7% at the 1-year follow-up, which aligns with the findings of Ueda et al. [15] who reported restenosis rates of 11.9% in the stenting group. These findings emphasize the ongoing concern of restenosis despite successful initial procedures and highlight the importance of monitoring patients post stenting. Our study also included a detailed analysis of lesion characteristics, such as lesion location and severity, which may provide valuable insights into the factors contributing to restenosis.

In terms of mortality, Stracke et al. [1] did not report 1-year mortality rates, as their study concentrated on the acute phase of stroke, where adverse events were similar between the stenting and medical treatment groups. However, our study identified a low mortality rate of 6.7% at 1 year, with a clear association between mortality and factors such as older age, a history of stroke, atrial fibrillation (AF), and rheumatic mitral valvular disease. Notably, Gong et al. [17] emphasized the significant influence of lesion characteristics, particularly hemodynamic impairment, on long-term outcomes following stent placement, which is consistent with our observation that patients with larger or more complex lesions tended to experience worse functional outcomes. Ueda et al. [15], while focusing on restenosis and long-term outcomes after balloon angioplasty and stenting, reported similar restenosis rates, however, did not provide mortality data at 1 year. Yu et al. [16], though concentrating on predictors of in-stent restenosis, did not place as much emphasis on mortality and functional status, instead focusing on procedural outcomes.

We believe that our study’s detailed mRS analysis provided deeper insight into the functional status of patients at the 1-year follow-up. It showed that the presence of comorbidities such as a prior stroke, AF, and rheumatic mitral disease was significantly associated with poorer functional outcomes. Additionally, larger infarct volumes or lesions located in critical vascular territories correlated with worse functional recovery. The relationship between higher mRS scores and mortality in our study further underscores the importance of pre-existing conditions and lesion characteristics in predicting long-term outcomes. These findings are consistent with Ueda et al. [15], which identified baseline patient characteristics as strong predictors of poor functional recovery, although their study did not report mRS scores specifically. Yu et al. [16] similarly noted that patients with a higher burden of comorbidities had worse functional recovery, though mRS scores were not a primary focus of their study. Gong et al. [17] further corroborated our findings by demonstrating that more severe hemodynamic impairment in lesions was linked to poorer functional outcomes, thus emphasizing the importance of lesion characteristics in predicting recovery after stenting.

Our study differs from previous studies in several ways. First of all, we aimed to provide a comprehensive evaluation of both short-term and 1-year outcomes following intracranial stenting, with a particular focus on the interplay between lesion characteristics, mortality, functional status, and factors contributing to poor outcomes. The differences observed between our study and others are likely due to variations in study designs, patient cohorts, lesion locations, and procedural focuses. One key strength of our study is its holistic approach to intracranial stenting outcomes, particularly in terms of 1-year mortality, functional status, and the role of lesion characteristics. Our cohort included a considerable number of patients who underwent intracranial stenting either for acute stroke unresponsive to mechanical thrombectomy or for symptomatic intracranial atherosclerotic stenosis, providing insights into both urgent and elective stenting procedures.

On the other hand, our study has several limitations. The absence of a control group of patients treated medically limits our ability to directly compare outcomes between stenting and medical management. The relatively low number of deaths also restricts the statistical power to identify strong predictors of mortality. Additionally, our findings may not be generalizable to other hospital settings, and the patient cohort, which primarily included those with acute stroke or symptomatic atherosclerotic stenosis, may introduce selection bias. Incomplete follow-up for some patients could also affect long-term outcome analysis. As a non-randomized study, unmeasured confounders may have influenced the results. Moreover, we did not explore other factors, such as genetic or procedural variations, that could impact outcomes.

## 6. Conclusions

Our findings suggest that the rates of death and restenosis are low in a 1-year follow-up after intracranial stenting. Functional dependence, advanced age, previous stroke, AF, and rheumatic mitral valve disease seem to be associated with death. Restenosis or urgency of the intervention does not affect mortality. Future studies, which include a large sample size of both urgent and elective stenting, participants with a greater level of functional independence or with diverse locations of atherosclerotic stenosis may explain the factors associated with mortality after stenting.

## Figures and Tables

**Table 1:** Baseline and follow-up characteristics of the patients.

		n	%
Location of target lesion			
	Right VA V4 branch	3	5.0
	Left VA V4 branch	19	31.7
	Right PCA	1	1.7
	Left PCA	0	0.0
	Basilar artery	17	28.3
	Right MCA	6	10.0
	Left MCA	5	8.3
	Right ICA lateral segment	8	13.3
	Right ICA petrous segment	1	1.7
1-year mortality			
	Absent	56	93.3
	Death from major stroke	2	3.3
	Death from MI	2	3.3
		Mean (SD.)	Median (Min–Max)
Age (year-old) (n = 60)		60.2 (10.8)	63 (35–81)
BMI (kg/m^2^) (n = 60)		29.1 (3.8)	28.4 (22.9–38.5)
Amount of contrast media (mL) (n = 60)		246.2 (34.3)	250 (200–300)
Diameter of stenosis (mm) (n = 60)		3.9 (0.5)	4 (2.5–4.5)
Length of stenosis (mm) (n = 60)		22.9 (5.5)	23.5 (15–39)
mRS score on admission (n = 60)		2.3 (0.7)	2 (1–5)
NIHSS score on admission (n = 7)		14.0 (3.7)	14 (10–20)
Duration between admission and intervention (min) (n = 7)		180.0 (29.4)	170 (150–240)
Stay at ICU (days) (n = 60)		3.0 (5.1)	1 (1–30)
Duration of hospitalization (days) (n = 60)		6.6 (6.4)	4 (2–30)

SD.: standard deviation VA: vertebral artery PCA: posterior cerebral artery MCA: middle cerebral artery MI: myocardial infarction BMI: body mass index ICU: intensive care unit.

**Table 2:** Categorical parameters of the patients at baseline and in the follow-up.

Categorical Parameters	n (%)
Age (≤65/>65-year-old)	42 (70)/18 (30)
Sex (female/male)	25 (41.7)/35 (58.3)
Smoking (absent/present)	29 (48.3)/31 (51.7)
T2D (absent/present)	33 (55)/27 (45)
HT (absent/present)	26 (43.3)/34 (56.7)
HF (absent/present)	52 (86.7)/8 (13.3)
Previous history of stroke (absent/present)	54 (90)/6 (10)
CAD (absent/present)	50 (83.3)/10 (16.7)
HL (absent/present)	27 (45)/33 (55)
Vasculitis (absent/present)	57 (95)/3 (5)
CKD (absent/present)	59 (98.3)/1 (1.7)
AF (absent/present)	49 (81.7)/11 (18.3)
Rheumatic mitral valve disease (absent/present)	58 (96.7)/2 (3.3)
Pretreatment with tPA (absent/present)	53 (88.3)/7 (11.7)
mRS (≤2/>2)	44 (73.3)/16 (26.7)
Type of aortic arch (I/II/III)	32 (53.3)/22 (36.7)/6 (10)
Site of entry (brachial/femoral)	7 (11.7)/53 (88.3)
Predilatation (absent/present)	3 (5)/57 (95)
Left ICA (absent/noncritical/critical)	32 (53.3)/22 (36.7)/6 (10)
Right ICA (absent/noncritical/critical)	44 (73.3)/13 (21.7)/3 (5)
Left VA (absent/noncritical/critical)	43 (71.7)/12 (20)/5 (8.3)
Right VA (absent/noncritical/critical)	49 (81.7)/3 (5)/8 (13.3)
Urgency of intervention (urgent/elective)	7 (11.7)/53 (88.3)
In-hospital mortality (absent/present)	59 (98.3)/1 (1.7)
Restenosis in 1st year (absent/present)	56 (93.3)/4 (6.7)
1-year mortality (alive/dead)	56 (93.3)/4 (6.7)

T2D: type 2 diabetes mellitus, HT: hypertension, HF: heart failure, CAD: coronary artery disease, HL: hyperlipidemia, CKD: chronic kidney disease, AF: atrial fibrillation, tPA: tissue plasminogen activator, ICA: internal carotid artery, VA: vertebral artery.

**Table 3:** Association of clinical parameters with 1-year mortality.

		Alive (n = 56)	Exitus (n = 4)	*p*
Age (years)		62 (35–81)	78.5 (71–81)	**<0.001 ^U^**
Age (>65-year-old)		14 (25)	4 (100)	**<0.001 ^f^**
BMI (kg/m^2^)		29.4 (22.9–38.5)	26.1 (23.4–29)	0.089 ^U^
Sex (female)		24 (42.9)	1 (25)	0.364 ^f^
Smoking		27 (48.2)	4 (100)	0.113 ^f^
T2D		25 (44.6)	2 (50)	0.999 ^f^
HT		30 (53.6)	4 (100)	0.126 ^f^
HF		6 (10.7)	2 (50)	0.082 ^f^
Previous history of stroke		3 (5.4)	3 (75)	**0.002 ^f^**
CAD		8 (14.3)	2 (50)	0.126 ^f^
HL		30 (53.6)	3 (75)	0.620 ^f^
Vasculitis		3 (5.4)	0 (0)	0.999 ^f^
CKD		1 (1.8)	0 (0)	0.999 ^f^
AF		8 (14.3)	3 (75)	**0.017 ^f^**
Rheumatic mitral valve disease		0 (0)	2 (50)	**0.003 ^f^**
Pretreatment with tPA		6 (10.7)	1 (25)	0.399 ^f^
mRS score (>2)		12 (21.4)	4 (100)	**0.004 ^f^**
mRS score		2 (1–4)	3 (3–5)	**0.001 ^U^**
Amount of contrast media		250 (200–300)	230 (200–300)	0.925 ^U^
Diameter of stenosis		4 (2.5–4.5)	3.3 (2.5–4.5)	0.132 ^U^
Length of stenosis		23.5 (15–39)	21.5 (17–30)	0.980 ^U^
Stay at ICU (days)		1 (1–25)	2 (1–30)	0.552 ^U^
Duration of hospitalization (days)		4 (2–30)	5.5 (3–30)	0.340 ^U^
Site of entry (femoral)		50 (89.3)	3 (75)	0.399 ^f^
Predilatation		53 (94.6)	4 (100)	0.999 ^f^
Type of aortic arch				0.414 ^ff^
	I	29 (51.8)	3 (75)	
	II	22 (39.3)	0 (0)	
	III	5 (8.9)	1 (25)	
Degree of stenosis (absent/noncritical/critical)				
	Left ICA	30 (53.6)/20 (35.7)/ 6 (10.7)		0.141 ^ff^
	Right ICA	41 (73.2)/12 (21.4)/3 (5.4)		0.999 ^ff^
	Left VA	40 (71.4)/11 (19.6)/5 (8.9)		0.999 ^ff^
	Right VA	45 (80.4)/3 (5.4)/8 (14.3)		0.999 ^ff^
Urgency of intervention (urgent)		6 (10.7)	1 (25)	0.399 ^f^
Restenosis in 1st year		4 (7.1)	0 (0)	0.999 ^f^

^f^ Fisher Exact Test (Monte Carlo), ^ff^ Fisher–Freeman–Halton test (Monte Carlo), ^U^ Mann–Whitney U test (Monte Carlo), data were demonstrated as n (%) or median (min–max). BMI: body mass index, T2D: type 2 diabetes mellitus, HT: hypertension, HF: heart failure, CAD: coronary artery disease, HL: hyperlipidemia, CKD: chronic kidney disease, AF: atrial fibrillation, tPA: tissue plasminogen activator, ICU: intensive care unit, ICA: internal carotid artery, VA: vertebral artery.

## Data Availability

The datasets generated during and/or analyzed during the current study are available from the corresponding author upon reasonable request.
